# Genome Editing in the Chicken: From PGC-Mediated Germline Transmission to Advanced Applications

**DOI:** 10.3390/ijms26199426

**Published:** 2025-09-26

**Authors:** Jiliang He, Ningkun Shi, Hongqin Yao, Juan Li, Yajun Wang, Jiannan Zhang

**Affiliations:** 1 Key Laboratory of Bio-Resources and Eco-Environment of Ministry of Education, College of Life Sciences, Sichuan University, Chengdu 610065, China; hejiliang@stu.scu.edu.cn (J.H.); 2023222040077@stu.scu.edu.cn (N.S.); 2021222040073@stu.scu.edu.cn (H.Y.); lijuanscuhk@163.com (J.L.); 2 Animal Disease Prevention and Green Development Key Laboratory of Sichuan Province, College of Life Sciences, Sichuan University, Chengdu 610065, China

**Keywords:** poultry, genome editing, PGCs, transgenic, CRISPR/Cas9

## Abstract

Avian genome editing has historically lagged behind mammalian research. This disparity is primarily due to a unique reproductive biology that precludes standard techniques like pronuclear injection. A pivotal breakthrough, however, came from the development of efficient in vitro culture systems for primordial germ cells (PGCs). This has established the chicken as a tractable and powerful model for genetic engineering. Our review chronicles the technological evolution this has enabled, from early untargeted methods to the precision of modern CRISPR-based systems. We then analyze the broad applications of these tools, which are now used to engineer disease resistance, enhance agricultural traits, and develop novel platforms such as surrogate hosts and oviduct bioreactors. Collectively, these advances have established PGC-based genome editing as a robust and versatile platform. Looking forward, emerging precision editors and the expansion of these techniques to other avian species are poised to drive the next wave of innovation in poultry science and biotechnology.

## 1. Introduction

The poultry industry is a cornerstone of global food security, supplying essential dietary protein through the production of meat and eggs [1]. Beyond their agricultural importance, chickens serve as an invaluable model organism for research in developmental biology and neuroscience, while probing their unique physiological characteristics presents challenges that demand precise genome editing technologies. The feasibility of this approach has been demonstrated by the successful production of transgenic chickens via germline transmission using primordial germ cells (PGCs) [2,3,4].

The generation of genome-edited avian species depends on two synergistic technologies: a vehicle for germline transmission and a tool for genome editing. The former involves the manipulation of germ cells to ensure that genetic modifications are heritable, while the latter utilizes modern molecular techniques for targeted DNA sequence modification [5]. In this review, we chronicle recent advances in chicken genome editing, discuss the practical applications of these resulting genetically modified organisms, and offer perspectives on future directions for this transformative field.

## 2. Strategies for Germline Transmission

Despite rapid advancements in genome editing technologies, their application in avian species has lagged significantly behind that in mammals. This disparity stems from substantial technical challenges posed by the structural complexity of avian fertilized eggs and fundamental developmental differences compared to mammalian embryos [6,7]. To circumvent these obstacles, research has shifted from manipulating single-cell zygotes to alternative approaches that leverage the germ cells abundant within the avian reproductive system. An early strategy involved direct embryo manipulation via shell windowing: a procedure encompassing embryo exposure, microinjection, and shell resealing to facilitate germline modification [8]. Building on such foundational methods, current avian germline transmission technologies can be broadly classified into two main approaches: conventional strategies not involving primordial germ cells (PGCs) and contemporary techniques centered on PGCs.

### 2.1. Non-PGC-Mediated Strategies

#### 2.1.1. Direct Embryo and Blastoderm Manipulation

Embryonic cells derived from avian eggs have been experimentally manipulated for decades through viral vector or plasmid delivery systems. The heterogeneous nature of these target cell populations has historically limited precise identification of functionally contributing cellular subtypes. A seminal breakthrough came from Marzullo, who demonstrated the principle of germline transmission by transplanting blastodermal cells between pigmented and non-pigmented chicken strains [9]. This foundational work paved the way for producing transgenic avians, particularly through the DNA microinjection methodology established by Love et al. This protocol involves microinjecting genetic constructs—such as plasmids, viral vectors, or transposon systems—directly into the blastoderm of embryos at the Eyal-Giladi and Kochav (EGK) stage X. At this stage, the blastoderm contains approximately 55,000 cells [10]. As a direct in vivo method, this technique allows genomic integration within the embryo, bypassing the complexities of in vitro cell culture [11]. Its operational simplicity made it a foundational technique in avian transgenesis.

Bosselman et al. demonstrated successful germline integration by microinjecting a replication-defective reticuloendotheliosis virus (REV) vector, ME111, into the blastoderm of early-stage chick embryos, which produced chimeric G0 males with vector provirus integrated into their gonadal tissues [12]. To mitigate host damage from viral vectors, subsequent research explored plasmid-based delivery, but these non-viral methods proved far less efficient. For instance, Naito et al. introduced plasmid DNA into fertilized egg germinal disks, though post-maturation analysis of 25 chickens revealed transgene presence in only one male’s blood sample and another’s semen [13]. Parallel investigations by Kino et al. employing histochemical staining combined with PCR and Southern blot analyses demonstrated transient nuclear retention and limited chromosomal integration of cytoplasmically injected DNA in fertilized avian ova [14].

Microinjection techniques remain under continuous optimization. Longmuir et al. developed a peptide/non-cationic lipid gene delivery system comprising three key components: a ‘condensed’ peptide segment with alternating hydrophobic and cationic amino acid residues, a ‘fusion’ peptide featuring membrane-insertion capability through amphiphilic helical domains, and a short-chain phosphatidylcholine derivative [15]. Notably, Polyethylene glycol (PEG) was conjugated to dioleoyl phosphatidylethanolamine via disulfide linkages within this formulation. The researchers systematically optimized this delivery platform, achieving robust transgene expression in chicken embryos via microinjection. This result confirmed the system’s functional efficacy [15].

#### 2.1.2. EG and ES Cell-Mediated Transmission

Stem cell-mediated germline transmission represents another key strategy in avian genetic engineering. This methodology involves the in vitro culture and genetic modification of pluripotent stem cells, which are subsequently reintroduced into a developing host embryo to generate chimeras.

Pioneering work by Park et al. established avian germline chimerism using embryonic germ (EG) cells derived from cultured PGCs on fibroblast feeder layers [3]. They later refined culture conditions for PGCs isolated from 5.5-day-old gonads (stage 28), using a medium supplemented with factors such as SCF, LIF, and bFGF to establish stable EG cell lines. These lines retained the molecular characteristics of both PGCs and undifferentiated stem cells [16]. Concurrently, Pain et al. identified and maintained putative avian embryonic stem (ES) cells from early embryonic disks using a culture system of trophic factors and an STO feeder layer [17].

Building upon these advancements, Van De Lavoir et al. developed an integrated strategy combining transient embryonic environmental manipulation with transgenic chicken embryonic stem (cES) cell introduction. By integrating a GFP-encoding transgene into cES cells and transplanting them into Stage VI-X (Eyal-Giladi and Kochav) recipient embryos, they achieved widespread somatic GFP expression, confirming successful germline transmission and tissue integration [18].

#### 2.1.3. Sperm- and Ovary-Mediated Transgenesis

The advancement of avian genome editing has driven innovation in pre-ovulatory germline delivery systems. Initial breakthroughs included testis-mediated gene transfer (TMGT) and transplanted transfected spermatogonial stem cells (TTSSCs) [19]. This approach uses the testicular microenvironment to incorporate foreign genetic material into sperm-producing cells. Lee et al. demonstrated interspecies testicular cell transplantation by injecting white-feathered broiler cells into flighted chicken seminiferous tubules, achieving 7.8% testicular chimerism [20]. Subsequent studies by Trefil et al. applied this technique to gamma-irradiated sterile roosters, restoring spermatogenesis in 50% of recipients [21]. They also reported that transplantation of primary germ cells expressing mCherry or GFP into the testes of G0 generation sterile roosters similarly restored spermatogenesis and efficiently produced mCherry or GFP-positive transgenic G1 offspring [22].

Direct testicular gene delivery has also demonstrated efficacy. Li et al. pioneered a direct intratesticular administration approach by injecting roosters with recombinant plasmids encoding green fluorescent protein (GFP), establishing a novel high-efficiency methodology for generating transgenic poultry [23]. This technique demonstrated robust germline transmission efficiency, with 56.5% of F1 and 52.9% of F2 progeny exhibiting stable transgene integration [23]. Similarly, Min et al. used direct testicular injection to deliver an antiviral transgene (EGFP-MMx), achieving testicular chimerism in 72.2% of treated roosters [24].

A more refined strategy, sperm transfection assisted genome editing (STAGE), involves the in vitro transfection of mature spermatozoa followed by artificial insemination. Harel-Markowitz et al. used a liposomal agent to transfect cockerel sperm with an eGFP plasmid, resulting in transgene expression in 89.5% of the offspring (17/19 chicks) [25]. Collares et al. refined this approach through sperm-mediated gene transfer (SMGT), employing dimethyl sulfoxide (DMSO)-treated sperm depleted of seminal plasma for EGFP plasmid delivery [26]. Their findings confirmed that the combined use of plasmid DNA-DMSO complexes and sperm washing protocols enables effective in vivo transfection, as evidenced by transgene expression in resultant embryos. The STAGE platform was subsequently adapted for precision editing with CRISPR/Cas9. Cooper et al. successfully used STAGE to generate GFP-knockout chickens and to induce targeted mutations in the doublesex and mab-3 related transcription factor 1 (*DMRT1*) gene [27].

While most strategies have targeted male germ cells, recent innovations have begun to explore the female germline. Jiang et al. developed an innovative laparoscopic technique for direct gene delivery [28]. They created an abdominal window to inject a liposomal GFP plasmid into immature ovarian follicles and then used artificial insemination to produce genome-edited offspring. This approach achieved a maximum transgenic efficiency of 12.1%, demonstrating scalable potential [28]. Future research will likely focus on improving the efficiency, predictability, and safety of these direct germline editing techniques. Extending these simplified methods to other poultry species where PGC culture is not yet established could dramatically accelerate the development of genetically engineered birds for both agricultural and research purposes.

### 2.2. PGC-Mediated Germline Transmission

#### 2.2.1. Biology and Characterization of PGCs

Germ cells serve as fundamental biological units for transmitting genetic information between generations and ensuring species survival [29]. PGCs are the embryonic progenitors of gametes, destined to undergo meiosis to form haploid sperm or oocytes [30]. While PGCs often remain quiescent during embryogenesis, the regulatory pathways governing their specification and differentiation exhibit significant variation across species [30]. In human developmental biology, PGCs represent the foundational undifferentiated stem cell population, showing superior germline competency compared to other pluripotent stem cell types including embryonic stem cells (ESCs) and embryonic germ cells (EGCs) [31]. Advancements in stem cell biology and differentiation methodologies have enabled the in vitro derivation of PGCs from pluripotent stem cell sources, offering new therapeutic potential for infertility [32]. Notably, in most vertebrate models, including zebrafish and mice, PGCs originate extragonadally from somatic lineages (somatic gonadal precursors, SGPs), necessitating directed embryonic migration to reach their functional niches [33].

Unlike most species, avian PGCs exhibit a unique biological characteristic: their migration to the germinal ridge occurs through the vascular system. Following Waldeyer’s initial identification of PGCs in 1870, research in birds revealed that these cells originate from the endodermal germinal wall, a discovery that led to the coining of the term “germ cells” [31]. In avian species, PGCs undergo multiple phases of proliferation and motile activity. During development at the Eyal-Giladi and Kochav (EGK) stage X, these cells accumulate in substantial quantities within the hypoderm and ectoderm layers of the zona pellucida [10]. At EGK-X stage, these cells initially anchor to the centrally expanding hypodermal layer within the zona pellucida, subsequently dissociating from the epiblast. The migratory trajectory of PGCs occurs within the germinal epithelium of avian embryos, commencing with passive diffusion followed by directional displacement along linear pathways from intermediate to anterior regions of the zona pellucida.

Following this initial displacement, PGCs establish adhesion to the basal lamina, a specialized extracellular matrix structure. Experimental evidence from Kang et al. demonstrates subsequent active recruitment of PGCs into the developing crescent region [34]. In vitro observations reveal pseudopod formation during migratory phases, suggesting amoeboid-like motility mechanisms. The vascular migration phase initiates at Hamburger-Hamilton (HH) stages HH9-10, reaches maximal intensity at HH12, and culminates in somatic epithelial infiltration at the germinal crest during stages HH15-18, as documented by Mathan et al. [35].

The characterization of cells maintained in vitro is critical for pluripotent cell studies. Jung et al. demonstrated that chicken PGCs could be identified using multiple labeling reagents, including antibodies against PAS, SSEA-1, and EMA-1, as well as lectins STA and DBA [36]. Co-staining with markers targeting SSEA-3, SSEA-4, and integrins α6 and β1 proved particularly effective for rapid PGC identification [36]. Subsequent characterization by Macdonald et al. revealed that cultured PGCs exhibited distinctive morphological features including large lipid vacuoles and glycogen accumulation [37]. These cells simultaneously expressed the stem cell marker SSEA-1, germ cell-specific proteins CVH and CDH, and pluripotency-associated genes (*MYC*, *KLF4*, *POUV*, *SOX2*, and *NANOG*) [37].

Motono et al. identified differential expression patterns in SSEA-1-positive populations, observing that *Blimp1*, *SOX2*, and *CXCR4* expression disappeared in SSEA-1-negative cells [38]. Their temporal analysis revealed diminished *CXCR4* expression following the circulatory phase of PGCs, indicating this chemokine receptor’s functional importance during PGC migration from the peripheral circulation to developing gonads [38]. This role was confirmed when CRISPR/Cas9-mediated mutation of the *CXCR4* gene severely impaired the migratory capacity of transplanted chicken PGCs [39].

#### 2.2.2. Sourcing and Isolation of PGCs

For generating germline chimeras, PGCs can be isolated from three primary sources: the early-stage germinal crescent, embryonic blood, and embryonic gonads. Although technically feasible, isolating PGCs from the germinal crescent (stages 4–8) is inefficient due to the low cell numbers. Consequently, current methodologies predominantly rely on two sources that offer higher yields: circulating PGCs harvested from embryonic blood (stages 13–15) and gonadal PGCs (gPGCs) isolated from primordial gonads after they have completed their migration (stages 27–29) [40]. For instance, Yu et al. achieved a 12.6% chimerism rate by transplanting blood-derived PGCs between chicken strains [41]. The versatility of this approach is further highlighted by successful interspecies transplantation, such as transferring pheasant PGCs into chicken embryos [42] or chicken PGCs into quail embryos [43], demonstrating their robust migratory and colonization capabilities. Comparative analyses have also shown that blood-derived PGCs may exhibit superior migration potential compared to those sourced from gonads [44].

In addition to identifying the optimal sources, various techniques have been developed to isolate and purify PGCs from contaminating somatic cells. These methods can be broadly classified into two strategies: antibody-independent physical separation and antibody-mediated cell sorting. The former approach leverages the unique physical properties of PGCs, such as their size and density, with techniques like density gradient centrifugation being widely used [45,46]. In contrast, antibody-mediated techniques like fluorescence-activated cell sorting (FACS) and magnetic-activated cell sorting (MACS) utilize specific cell surface markers to achieve high-purity PGC isolation [47,48].

#### 2.2.3. In Vitro Cultivation of PGCs

The ability to culture PGCs in vitro is a cornerstone of modern avian genetic engineering. While short-term culture is sufficient for simple transfers, long-term culture systems are essential for performing complex genomic modifications and selecting successfully edited cell clones before transplantation. Early PGC cultures relied on feeder layer cells and could only be maintained in vitro for short durations. Karagenç et al. demonstrated the isolation of approximately 150 PGCs per embryo by culturing cells dispersed from stage IX-XIII clear zones on STO feeder layers [49]. In 1997, Chang et al. used germinal ridge stroma cells (GRSCs) as feeders in a medium supplemented with insulin-like growth factor 1 (IGF-1), FGF2, LIF, and serum, achieving five-day maintenance of chicken PGCs [50].

A significant breakthrough came from Van De Lavoir et al., who established a long-term PGC culture system. By employing STO or buffalo rat liver (BRL) feeder cells in knockout DMEM medium enriched with stem cell factor (SCF) and human recombinant fibroblast growth factor (FGF), they established sustained in vitro propagation of chicken PGCs [51]. Miyahara et al. further optimized culture conditions using BRL feeder cells in basic medium containing bFGF alone or combined with SCF, producing 6% chimeric males from PGC-like cells and demonstrating successful germline transmission through three progeny generations [52]. The evolution of feeder layer systems subsequently shifted toward avian-derived substrates. Ji et al. achieved five-week in vitro cultivation of PGCs by transplanting 5.5-day embryonic germinal ridge cells onto chicken embryonic fibroblasts (CEFs) [53]. Further innovations include the use of indirect co-culture systems, where porous membrane inserts facilitate nutrient exchange while simplifying subculturing and preventing direct cell-to-cell contact [54,55]. Most recently, Szczerba et al. developed a feeder-dependent system using chick embryo-derived cells and KAv-1 medium, enabling simultaneous proliferation of cPGCs and gPGCs with successful gonadal colonization in recipient embryos [56].

However, feeder-dependent cultures have significant drawbacks. They often rely on high concentrations of fetal bovine serum (FBS), making them costly, and the feeder layer complicates applications like transfection. Furthermore, freshly isolated PGCs exhibit slow initial proliferation, and the extended cultivation required often leads to substantial cell loss via apoptosis, markedly reducing overall efficiency [55]. This has driven the development of feeder cell-free systems.

Whyte et al. demonstrated that a combination of FGF2, insulin, and Activin A could support PGC self-renewal by activating the ERK1/2, Akt, and SMAD3 signaling pathways [57]. This feeder-free methodology has been successfully adapted to derive germline chimeras in multiple native chicken breeds [58,59]. Comparative analysis revealed frozen PGCs exhibit enhanced pluripotency and elevated expression of PGC-specific markers (*SSEA-1*, *NANOG*, *OCT4*, *DAZL*, and *CVH*) relative to freshly isolated counterparts [60]. Niu et al. employed RNA sequencing to demonstrate differential gene expression profiles between HIS and FACS culture systems in cellular proliferation, pluripotency regulation, and adhesion mechanisms [61]. Both systems effectively preserved reproductive marker expression, pluripotent potential, and characteristic glycogen granule accumulation in chicken PGCs [61]. Driven by advantages in efficiency and reproducibility, feeder-free methodology is rapidly becoming the preferred strategy.

The development of defined media has enabled a systematic elucidation of the molecular machinery governing PGC self-renewal and survival. This machinery is controlled by a core set of signaling pathways that regulate the fundamental cellular processes of proliferation, apoptosis, and pluripotency maintenance (Table 1).

The FGF and PI3K/AKT/mTOR axis serve as the primary engine for PGC proliferation and survival. Basic fibroblast growth factor (FGF2) acts as a potent mitogen, activating multiple downstream cascades, including the Ras/Raf/MEK/ERK and PI3K pathways, which are essential for driving cell cycle progression [57,62,63]. Insulin, another critical component, functions synergistically with FGF2. Its absence from the culture medium leads to a complete cessation of PGC proliferation, a decline in pluripotency marker expression, and the induction of apoptosis [64]. Mechanistically, insulin binds to its receptor to activate the PI3K/AKT/mTOR pathway. This activation is crucial for upregulating downstream targets that promote cell growth and survival while suppressing apoptosis [65]. The PI3K/AKT pathway is also intrinsically linked to the maintenance of pluripotency, as its activation supports the expression of core transcription factors such as NANOG and SOX2. Together, FGF2 and insulin provide the robust pro-growth and anti-apoptotic signals necessary to sustain PGC expansion in vitro [57,64,65,66].

The Transforming Growth Factor-β (TGF-β) superfamily plays a more nuanced role, orchestrating the delicate balance between self-renewal and differentiation. This family includes two key branches with distinct functions in PGC biology. The Activin/Nodal branch, signaling through the phosphorylation of SMAD2/3, is essential for maintaining the self-renewing state of chicken PGCs in defined, clonal-growth conditions [57]. In vivo and in vitro analyses confirm that chicken PGCs express the requisite receptors (e.g., ACVR2A/B, ALK4/5/7) and exhibit nuclear localization of phosphorylated SMAD2, indicating an active pathway [57,67,68]. In contrast, the Bone Morphogenetic Protein (BMP) branch, which signals via SMAD1/5/8 phosphorylation, is more centrally involved in the initial specification of PGCs from the epiblast during embryonic development [69,70,71,72]. While BMP4 can substitute for Activin A in supporting the proliferation of chicken PGCs in non-clonal culture conditions, its primary role appears to be in fate determination rather than the stable maintenance of the self-renewing state [57].

The WNT signaling pathway functions as a critical modulator of PGC development and self-renewal [71,73]. Transcriptomic studies have shown that WNT signaling is downregulated upon insulin withdrawal, indicating its role in proliferation. Insulin appears to promote the expression of key pathway components like Wnt-5B, which in turn supports PGC self-renewal [64]. Furthermore, targeted activation of the WNT pathway has been shown to significantly improve the efficiency of inducing PGC-like cells from pluripotent stem cells, highlighting its importance in establishing the germline program [69]. While not a primary component of the minimal self-renewal cocktail defined by Whyte et al. [57], WNT signaling is evidently a crucial supportive pathway that works in concert with the FGF, PI3K/AKT, and TGF-β axes to fine-tune PGC fate.

Building upon the mechanistic understanding of core signaling pathways, research has continued to refine culture conditions. Studies have shown that supplementation with fetal calf serum (FCS), insulin–transferrin–selenium (ITS), or Knockout Serum Replacement (KOSR) can significantly improve PGC growth and proliferation [74,75]. A more recent and powerful strategy uses small-molecule inhibitors to actively suppress cellular processes detrimental to long-term culture, such as apoptosis. This approach represents a paradigm shift from merely providing pro-growth signals to actively neutralizing specific failure points within the cellular system. The myosin II inhibitor blebbistatin has proven exceptionally effective in this regard, significantly enhancing both the survival and proliferation rate of cultured PGCs [76]. Other small molecules are also being explored to optimize PGC culture and induction. For instance, antagonists of the aryl hydrocarbon receptor (AHR), such as α-naphthoflavone, have been reported to promote the expansion of the undifferentiated PGC population [77]. In the context of generating PGCs de novo from pluripotent cells, a cocktail of small molecules that activates the WNT and cAMP pathways while simultaneously inhibiting the MAPK pathway has been shown to significantly increase induction efficiency. These combinatorial approaches, which fine-tune multiple signaling nodes simultaneously, hold great promise for developing highly efficient and reproducible protocols for both culturing and deriving avian PGCs [69].

Despite significant progress, several critical bottlenecks continue to challenge the long-term culture and application of avian PGCs. Addressing these issues is paramount for realizing the full potential of this technology.

Apoptosis remains a major hurdle, but it is more than a simple technical problem. While excessive apoptosis reduces cell yields and overall efficiency, it is also a fundamental biological quality control mechanism. PGCs are the custodians of the germline, and they are hardwired to preserve genomic integrity at all costs. PGCs and somatic cells respond to cellular stress or DNA damage in fundamentally different ways. Under such conditions, PGCs are profoundly more likely to initiate apoptosis than to attempt DNA repair. This strategy contrasts sharply with that of somatic cells, which prioritize repair to ensure survival [78]. Upon detecting DNA damage, PGCs rapidly downregulate pluripotency genes like *NANOG* and upregulate pro-apoptotic genes such as *FAS* and *BAK1*, effectively eliminating themselves to prevent the transmission of mutations. This “guardian of the genome” function, while essential in vivo, creates a central paradox for in vitro culture, where the artificial environment itself can be a source of stress. The use of anti-apoptotic agents like blebbistatin is necessary to achieve sufficient cell numbers for genetic engineering, but this intervention must be carefully balanced to avoid the inadvertent propagation of cells with genomic abnormalities that would have otherwise been eliminated.

The challenge of species- and strain-specificity poses a significant barrier to the broad application of PGC technology. The culture protocols optimized for chickens are not universally applicable. A striking example is found in the goose, where PGC self-renewal is dependent on BMP4 and is actively inhibited by Activin A—the exact opposite of the core requirement in chickens [79,80,81]. This demonstrates a fundamental divergence in TGF-β superfamily signaling between avian species. This specificity extends even to the strain level within a single species. For example, Hubbard strain chicken PGCs show superior growth in an ovotransferrin-enriched medium (OTM), whereas Bovans strain PGCs thrive in a medium supplemented with chicken serum (CSM) [82]. These findings dismantle the notion of a single “avian model” for PGC culture. Consequently, extending PGC-based technologies to other important poultry species or to endangered birds is not straightforward. The process will likely require bespoke research and development for each species and potentially each distinct genetic line. This reality significantly increases the complexity and cost of such endeavors.

The risk of genomic instability during prolonged culture presents a crucial trade-off. While long-term maintenance is a prerequisite for complex, multi-step genome editing, it increases the likelihood of accumulating genetic and epigenetic aberrations that can compromise the germline competence of the cells [82]. The robust proliferation of PGCs can diminish over time, and their ability to contribute to the germline has been observed to decrease with extended passage [83]. The interventions used to overcome the apoptosis bottleneck may inadvertently select for cells that have lost their stringent quality control mechanisms, potentially leading to aneuploidy or other mutations. Therefore, the goal of long-term culture is not merely proliferation, but high-fidelity proliferation. Future protocols must incorporate routine and rigorous quality control checkpoints, such as karyotyping, targeted gene sequencing, and epigenetic profiling, to ensure the genomic integrity of PGC lines before their use in creating transgenic animals.

Advancements in the in vitro culture of PGCs have been foundational to the recent progress in avian genome editing. The evolution from feeder-dependent to defined, feeder-free systems has not only improved efficiency and reproducibility but has also unveiled the complex network of signaling pathways governing PGC fate. However, this deeper understanding has simultaneously highlighted the profound challenges posed by species-specific signaling requirements, the dual nature of apoptosis as both a technical hurdle and a biological safeguard, and the persistent risk of genomic instability during prolonged culture. The current reliance on well-established chicken PGC systems has concentrated most genetic engineering efforts on this single species, making it the primary model for both biological research and agricultural applications while leaving other important avian species behind.

#### 2.2.4. Transfection of PGCs

The transfection efficiency of PGCs remains a critical limiting factor in avian genome editing applications, with key determinants including transfection reagents, cellular status, and plasmid design. Early work by Hong et al. established that enhanced DNA uptake in chicken PGCs through electroporation optimization, revealing that dimethyl sulfoxide (DMSO) supplementation significantly improved germline-competent PGC (gPGC) transfection efficiency compared to liposomal methods [84]. Oishi et al. later developed a density gradient centrifugation method that yielded a sevenfold increase in transient transfection efficiency [85]. Comparative studies have systematically evaluated purification–transfection combinations. Puchta et al. identified Percoll density gradient centrifugation coupled with electroporation as optimal, achieving high transfection efficiency compared to alternative methods using ACK lysis or trypsinization [44]. Building upon this, Zou et al. recently demonstrated that systematic optimization of electroporation parameters, specifically pulse voltage and duration, could further elevate the transfection efficiency to over 70% while maintaining high cell viability [86].

Recent developments in liposomal technology have shown comparable efficacy, with Sawicka et al. reporting 67% efficiency using transposon vectors at optimized cell densities (5 × 10^5^ cells/mL) [87]. Watanabe et al. further quantified commercial reagent performance under heparin-free conditions, documenting 49.02 ± 10.54% efficiency for Lipofectamine™ 2000 versus 12.52 ± 1.05% for Lipofectamine™ 3000 [88]. While electroporation has historically demonstrated superior transfection rates, a recent comparative study by Meng et al. established that adenoviral vectors are even more effective, achieving up to 90% efficiency without the significant reduction in cell viability observed with electroporation [89]. However, the operational simplicity and cost-effectiveness of liposomal systems continue to drive their widespread adoption in avian PGC research, reflecting a common trade-off between maximal efficiency and practical applicability (Table 2). Therefore, future research should focus on developing novel transfection strategies that combine the convenience and low equipment dependency of chemical reagents with the high efficiency of electroporation or adenoviral vectors, aiming to fundamentally enhance the overall efficacy of the genome editing workflow.

#### 2.2.5. Cryopreservation of PGCs

The long-term storage of PGCs through cryopreservation is essential for biobanking and genetic resource management. The first successful cryopreservation protocol was developed by Naito et al. in 1994, using 10% DMSO and a slow-cooling method [90]. While these thawed PGCs could form germline chimeras, subsequent studies confirmed that freeze–thaw cycles significantly reduced cell survival (to ~46.5%) and diminished their functional capacity [91,92]. Work by Moore et al. demonstrated that PGCs isolated from Stage 27 gonads maintained viability following cryoprotectant-assisted freezing and resuscitation protocols, with post-thaw survival rates exceeding 50% [93].

To improve outcomes, research has focused on optimizing both methods and cryoprotectants. Aseptic vitrification has been shown to be superior to slow freezing, yielding better cell migration and colonization efficiency post-thaw [94]. The choice of cryoprotectant is also critical; 10% ethylene glycol (EG) was found to be more effective than equivalent concentrations of DMSO [93] and reducing DMSO concentration to 5% in combination with 20% fetal bovine serum also improved viability [95]. Recent innovations by Hamai et al. introduced propylene glycol (PG) as an alternative cryoprotectant, achieving high post-thaw recovery rates while maintaining germline potential [96]. Their comparative study of 7.5% PG versus 5% DMSO formulations revealed equivalent preservation efficacy, suggesting viable alternatives to traditional cryoprotectants [96].

## 3. Genome Editing Methodologies

While germline transmission provides the cellular vehicle for genetic inheritance, genome editing technologies offer molecular tools for its modification. Conventional approaches, such as viral transduction or transposon-mediated delivery, have been historically employed but are limited by their lack of target specificity, leading to random genomic integration [97]. This untargeted insertion of exogenous DNA risks chromosomal disruption via insertional mutagenesis, restricting the practical applications of the resulting organisms. The advent of programmable nucleases has revolutionized precision genome engineering through site-specific induction of double-strand breaks (DSBs), which are subsequently repaired via two distinct pathways: homology-directed repair (HDR) and non-homologous end joining (NHEJ). These repair mechanisms facilitate targeted genomic modifications including gene disruption, insertion, and correction. NHEJ employs enzymatic machinery for direct ligation of DNA termini, whereas HDR utilizes homologous templates to guide precise repair [98]. Contemporary genome-editing platforms predominantly employ zinc finger nucleases (ZFNs), transcription activator-like effector nucleases (TALENs), or the CRISPR/Cas9 system as core tools for targeted genetic manipulation [99]. This section analyzes these advanced technologies and their implementation in avian cellular systems.

### 3.1. Untargeted Genome Editing

#### 3.1.1. Retroviral Vectors

Viral vectors are a well-established tool for transgenesis. Retroviruses mediate untargeted genomic integration by inserting a transgene flanked by long terminal repeats (LTRs). This process is coordinated by three core genes: gag (encoding structural proteins), pol (encoding enzymes like reverse transcriptase and integrase), and env (mediating host cell entry). To ensure safety, modern replication-defective retroviral systems express these genes separately, preventing their packaging into new infectious particles [100].

The pioneering method for avian transgenesis involved injecting retroviral vectors into unincubated fertilized eggs. Early work by Salter et al. used two main viral systems, reticuloendotheliosis virus (REV) and avian leukemia virus (ALV), to demonstrate that microinjection into the blastoderm could result in germline integration, as confirmed by restriction enzyme analysis of the offspring [101,102]. Building on this, Bosselman et al. engineered a replication-defective REV vector carrying the chicken growth hormone gene. Detection of the vector and transgene expression in multiple tissues of G0 animals provided conclusive evidence of successful transduction in embryonic lineages [103]. Later, Kamihira et al. used a retroviral vector to create transgenic chickens that produced a recombinant antibody (scFv-Fc fusion protein) in both their serum and eggs, demonstrating stable, long-term expression [104].

#### 3.1.2. Lentiviral Vectors

A major limitation of standard retroviral vectors is the progressive silencing of transgene expression during development. Lentiviruses, a subclass of retroviruses, overcome this by maintaining transcriptional activity throughout embryogenesis and possessing the ability to integrate into the genome of non-dividing cells. These features have made them a valuable tool for avian transgenesis.

Chapman et al. created a comprehensive fluorescent reporter system in avian embryos by using a lentiviral vector to achieve ubiquitous eGFP expression [105]. McGrew et al. quantified the efficiency of this approach, finding that 70% of G0 birds contained the vector sequence and that the transgene was transmitted to offspring at rates of 4–45%, with stable inheritance through the G2 generation [106]. Lentiviral vectors have also been used to achieve neuron-specific GFP expression in transgenic quail for in vivo imaging [107] and to generate transgenic chickens and ducks by co-delivery with PGCs or direct embryonic injection [108,109].

#### 3.1.3. Transposon Systems

Transposon-based systems offer a non-viral method for untargeted genome editing. These systems typically use a transposase enzyme that recognizes terminal repeat (TR) sequences flanking a transgene, excises the cassette, and pastes it into a new genomic location. The most common transposon systems used for genome engineering are piggyBac [110], Sleeping Beauty, and Tol2 [111].

Sherman et al. successfully demonstrated mariner element transposition in chicken embryos through cytoplasmic microinjection of fertilized egg germinal disks [112]. Their findings revealed mariner integration in over 20% of embryos, with subsequent germline transmission efficiency reaching nearly 30% in offspring from a transgenic rooster [112]. Tyack et al. employed the Tol2 transposon system to integrate CAGGS promoter-driven EGFP constructs in both in vitro cell cultures and chicken embryos [113]. Through co-transfection of transposon vectors with transposase-encoding plasmids into early embryonic precursor mesoderm, they established transgene integration capacity, though no successful individual-level reports emerged from this work [113]. In a subsequent study, Tyack et al. achieved transgenic progeny production using Lipofectamine 2000-mediated delivery of Tol2 transposon/transposase complexes, demonstrating reporter gene expression and providing a retroviral-free methodology for avian transgenesis [114]. The piggyBac system has been particularly effective when combined with PGCs, achieving an average germline transmission efficiency of 95.2% and leading to stable transgene expression in the offspring [115]. It has also been used for direct transfection of stage X embryos, resulting in stable GFP expression across multiple tissue types [116].

### 3.2. Targeted Genome Editing

#### 3.2.1. Nuclease-Based Platforms: ZFNs, TALENs, and CRISPR

Zinc finger nucleases (ZFNs), the first targeted genome-editing tools developed in the early 21st century, comprise two functional components: (1) an array of 3–6 zinc finger proteins, each recognizing specific three-base pair sequences, and (2) the restriction endonuclease FokI, which requires dimerization to execute DNA cleavage [99]. TALENs have a similar architecture, but their DNA-binding domain is composed of TALE repeats, where specific repeat-variable di-residues (RVDs) recognize individual DNA bases (G, A, C, or T), allowing for more flexible targeting [117]. For both ZFNs and TALENs, two individual proteins must bind to opposite DNA strands, allowing their FokI domains to dimerize and create a targeted DSB [118]. Park et al. successfully employed TALENs to induce a nucleotide deletion mutation in the *OVA* (ovalbumin) gene within PGCs, resulting in the functional loss of the chicken *OVA* gene and subsequent generation of *OVA* mutant progeny [119]. Taylor et al. demonstrated that TALEN-mediated homology-directed repair at the *DDX4* locus on the Z chromosome achieved an efficiency of 8.1%, while a single TALEN pair generated a 30 kb deletion spanning the entire *DDX4* locus [120].

The CRISPR/Cas9 system, originally a bacterial adaptive immune system, has become the most widely used genome-editing tool. In this system, the Cas9 nuclease is directed to a specific 20 bp DNA target by a synthetic single guide RNA (sgRNA). The sgRNA is a fusion of two natural RNAs, crRNA and tracrRNA, that are essential for guiding Cas9 to its target [118,121,122,123]. Once bound, Cas9 creates a DSB, which is then repaired by non-homologous end joining (NHEJ) or homology-directed repair (HDR) [124].

The combination of CRISPR technology with refined PGC culture systems has accelerated avian genome engineering. Oishi et al. demonstrated over 90% editing efficiency when targeting the ovalbumin and ovomucoid genes in cultured PGCs [125]. This approach has been used to engineer the chicken immunoglobulin locus without affecting egg production [126] and to knock down the *Stra8* gene in both DF-1 cells and ESCs [127]. The versatility of CRISPR/Cas9 has been further demonstrated by its use in creating chickens with a sex-linked GFP marker for embryonic sexing [128], achieving dual fluorescent knock-in at the CVH locus with 82% efficiency [129], and editing the TYRP1 gene to alter feather color in regenerated chickens [130]. While all three technologies are powerful, CRISPR offers significant advantages in ease of use and cost, as it only requires the design of a short sgRNA rather than engineering complex DNA-binding protein domains. This has made it the dominant platform for targeted genome editing.

#### 3.2.2. Advanced CRISPR-Based Technologies

The core CRISPR system has been adapted into a diverse molecular toolbox. To address the critical issue of off-target effects, early optimization efforts focused on the rational design of high-fidelity (HF) SpCas9 variants. By reducing non-specific binding to the non-targeted DNA strand, variants like eSpCas9 and SpCas9-HF1 significantly improved specificity [131]. These high-fidelity nucleases have been successfully used in chickens to achieve precise large-segment deletions with minimal off-target effects, as demonstrated in the study of blue eggshell formation [132], and are now becoming the standard for applications requiring high precision. Advancing beyond this iterative refinement of natural enzymes, the frontier of CRISPR tool engineering is being rapidly reshaped by artificial intelligence (AI). A recent landmark study demonstrated this by using a large language model, trained on extensive metagenomic data, to design a novel editor, OpenCRISPR-1, completely de novo [133]. This AI-generated nuclease exhibits on-target activity and specificity comparable to human-engineered high-fidelity editors, despite being hundreds of mutations away from any known natural sequence. Such generative and predictive AI approaches are overcoming the throughput limitations of traditional protein engineering, enabling the rapid design of bespoke nucleases for advanced applications [134]. Although these AI-designed editors have yet to be deployed in avian systems, they represent a transformative future direction for poultry genome engineering.

Researchers engineered the Cas9 protein into a “dead” Cas9 (dCas9) variant by deactivating its nuclease domains. This dCas9 variant can bind to target DNA sequences without inducing cleavage [135]. When fused to transcriptional effectors, such as the repressor domain KRAB or the activator domain VP64, dCas9 enables programmable gene silencing (CRISPRi) or activation (CRISPRa), respectively [136,137]. These systems have proven to be robust platforms in avian models. For instance, they have been successfully applied to study transcriptional regulation in chicken DF-1 cells [138] and to dissect the function of cis-regulatory elements. Notably, beyond analyzing promoters [74], CRISPR-mediated activation has been powerfully employed to systematically characterize transcriptional enhancers across the chicken genome [139]. Despite these advances, comprehensive applications in avian models are still emerging. Nevertheless, the extensive deployment of CRISPRa/i in mammalian systems provides a clear roadmap for future poultry research. In those systems, CRISPRa/i has been instrumental in regulating endogenous pluripotency factors to modulate cell fate. For example, targeting the EEA motif and the promoter of the miR-302/367 cluster with CRISPRa enhanced the reprogramming efficiency of fibroblasts and human lymphoblastoid cell lines, accelerating the formation of induced pluripotent stem cells (iPSCs) [140]. Similarly, upregulating the neurogenic differentiation factor 1 (NEUROD1) gene in iPSCs via a dSpCas9-VPR activator induced neuronal differentiation within just 7 days [141]. Furthermore, a CRISPRi screen revealed that inflammatory signatures activated by NF-κB are regulated by STAT3 [142]. These powerful examples underscore the immense potential of CRISPRa/i for precisely controlling gene expression to investigate developmental biology, immunology, and key production traits in avian species.

Other Cas enzymes have expanded the molecular toolbox beyond conventional DNA editing. The Cas12a (formerly Cpf1) nuclease, for instance, exhibits a “collateral cleavage” activity on single-stranded DNA, a feature that has been widely repurposed for molecular diagnostics [143]. In avian research, this has led to the development of rapid, sensitive, and on-site detection platforms. These CRISPR-Cas12a-based assays, often combined with isothermal amplification methods like RPA or LAMP, have been used to detect pathogens such as avian influenza virus (AIV), including specific subtypes like H5, and various duck viruses [144,145,146]. Beyond pathogen detection, this technology has also been creatively applied to tasks such as the rapid genetic sexing of chickens [147]. In addition to its primary role in diagnostics, Cas12a has also been used for genome engineering in avian systems, such as in drug-inducible PGC ablation systems [148]. Similarly, the RNA-guided, RNA-targeting Cas13 enzymes offer dual functionality in avian research [149]. Their ability to directly target and degrade viral RNA has been leveraged for antiviral defense, significantly reducing influenza A virus titers in chicken cells [150]. Furthermore, leveraging its collateral RNA cleavage activity, Cas13a has been harnessed to create rapid and visual detection methods for RNA viruses like AIV [146].

While these nuclease-based methods are precise, the induction of DSBs always carries a risk of off-target mutations. To address this, various methods of guided RNA modification have been devised, including GC content, sgRNA length, truncated sgRNA, and chemical modification [151]. Even more precise technologies have been developed. Base editors are fusion proteins that combine a Cas9 nickase (which cuts only one DNA strand) or dCas9 with a DNA deaminase enzyme. These tools perform direct, single-nucleotide conversions without creating a DSB. Cytosine base editors (CBEs) convert C-G base pairs to T-A, while adenine base editors (ABEs) convert A-T pairs to G-C, together enabling all four possible transition mutations [152,153]. Prime editors are even more versatile. They consist of a Cas9 nickase fused to a reverse transcriptase enzyme. They are guided by a prime editing guide RNA (pegRNA) that not only specifies the target site but also contains a template for the new genetic information. This system can install all 12 possible base-to-base conversions, as well as small insertions and deletions, all without creating a DSB [154].

These precision editors have proven highly effective in vivo, but their application in avian species is still emerging. Lee et al. found that base editing is less efficient in avian PGCs than in somatic cells, likely due to high expression of base excision repair (BER) genes in germ cells that counteract the editing process [155]. The key element of BE4max, cytosine deaminase (APOBEC), was codon-optimized for chicken, which improved the base editing efficiency by about 10.4% [156]. Atsuta et al. recently provided the first proof-of-concept for prime editing in chicken cells, successfully correcting a reporter gene and introducing a stop codon into the endogenous *DDX4* locus in both fibroblasts and PGCs [157]. These findings highlight both the unique challenges and the immense potential of precision editing in avian biology.

**Figure 1:**
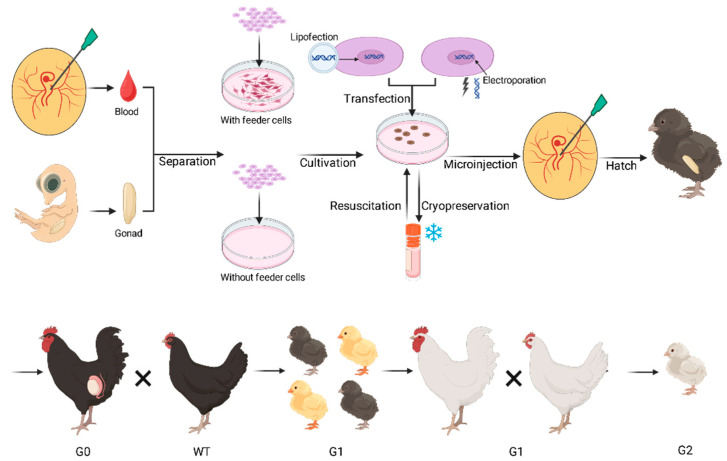
Schematic of the PGC-mediated genome-editing workflow in poultry. The process begins with the isolation of primordial germ cells (PGCs) from embryonic blood or gonads. These cells are then cultured and genetically modified in vitro using genome-editing tools such as CRISPR/Cas9. The engineered PGCs are subsequently microinjected into the vascular system of a recipient embryo to generate a germline chimera. Following maturation and breeding, this process leads to the production of genome-edited offspring for various applications.

The state-of-the-art approach to avian genome editing synthesizes the biological utility of PGCs with the molecular precision of programmable nucleases. This synergy allows for the in vitro engineering of the germline, followed by transplantation into a recipient embryo to create transgenic founders, a complete process illustrated schematically in Figure 1.

## 4. Applications of Genome Editing in Poultry

Advances in avian cell biology and genome editing have enabled the creation of genetically engineered birds, thereby validating the efficacy of these new molecular tools. These efforts are a prerequisite for translating research into commercial applications. In intensive poultry farming, infectious disease outbreaks pose a catastrophic economic risk, making disease resistance a primary research objective. Concurrently, enhancing production traits, such as muscle growth and egg characteristics, offers significant commercial value. The development of specialized animal models, including sterile avian hosts, accelerates research and reduces costs by improving the efficiency of generating transgenic offspring. Finally, the unique ovipositional biology of birds makes them ideal candidates for use as living bioreactors for producing therapeutic proteins. This section reviews four major domains of avian genome editing applications: enhancing disease resistance, improving agricultural production, developing novel animal models, and engineering avian bioreactors.

### 4.1. Engineering Disease Resistance

The most critical application of avian genome editing is the development of disease-resistant poultry. Viral infections can cause devastating losses and, in some cases, pose zoonotic risks to human health. A notable example is the emergent H7N9 subtype avian influenza virus, which demonstrates pandemic potential in human populations. Initial human cases were identified in early 2013, with mortality rates exceeding 30% by June of that year. The case fatality ratio exhibited a sustained upward trajectory as surveillance systems continued to detect new infections, prompting the World Health Organization (WHO) to designate H7N9 as an “exceptionally dangerous pathogen for humans” [158]. All avian influenza viruses (AIV) belong to type A, which has a broad host range that includes birds, swine, horses, and humans [159,160].

Several strategies have been employed to engineer influenza resistance. Lyall et al. generated transgenic chickens expressing a short hairpin RNA (shRNA) that acts as a decoy, targeting a conserved region of the influenza A virus polymerase to inhibit its function. While these transgenic chickens still succumbed to high-dose viral challenge, they did not transmit the virus to co-housed wild-type chickens, effectively breaking the chain of infection [161]. Similarly, Byun et al. created transgenic chickens expressing the 3D8 single-chain antibody (scFv), which possesses nuclease activity against viral genomes. Like the decoy-expressing chickens, these birds were not protected from direct infection but showed significantly reduced viral shedding, preventing transmission to contact birds [162,163]. A more direct approach was taken by Idoko-Akoh et al., who used CRISPR/Cas9 to edit the host factor ANP32A, a protein essential for influenza virus replication. Chickens with a targeted three-nucleotide substitution in ANP32A were completely resistant to infection, while chickens with a full gene knockout showed highly restricted viral replication [164].

Genome editing has also been successfully applied to combat other major poultry viruses. Avian leukosis virus subgroup J (ALV-J), which causes tumors and immunosuppression, enters host cells by binding to the chNHE1 receptor. Research identified a single amino acid, tryptophan-38 (Trp38), in the chNHE1 protein as being indispensable for viral entry [165]. Building on this, two independent groups, Koslová et al. and Hellmich et al., used CRISPR/Cas9 in PGCs to precisely delete the three nucleotides coding for Trp38 and showed the resulting chickens were completely resistant to ALV-J infection, and this resistance was stably inherited in commercial poultry lines [166,167].

For Marek’s disease (MD), a herpesvirus-induced cancer, research has focused on targeting the virus itself. CRISPR/Cas9 has been used to generate knockout mutants of viral microRNAs (miRNAs) essential for the viral life cycle [168], to inhibit the production of infectious MDV-2 virions from latently infected cell lines [169], and to create transgenic chickens that express both Cas9 and a guide RNA targeting the essential viral gene *ICP4*. When challenged with MDV, these transgenic chickens showed significantly reduced viral replication [170]. The key studies discussed above, which have successfully engineered resistance to major viruses such as influenza, ALV-J, and MDV, are summarized in Table 3. While genome-edited chickens resistant to other major viruses like Newcastle disease virus (NDV) or infectious bursal disease virus (IBDV) have not yet been reported, CRISPR-based approaches represent a promising strategy for future vaccine development and resistance engineering [171].

### 4.2. Enhancing Agricultural Production Traits

Beyond disease resistance, genome editing is being used to improve key production traits. A major target for enhancing muscle growth is myostatin (*MSTN*), a negative regulator of skeletal muscle development [172,173]. Xu et al. used an adenovirus vector to deliver CRISPR/Cas9 directly into the leg muscle of neonatal chicks, successfully knocking out the *MSTN* gene and altering gene expression pathways related to muscle development [174]. Lee et al. applied a similar CRISPR-based approach to quail embryos, generating homozygous mutant quail with significant muscle hyperplasia and increased body weight [175]. Alternatively, Bhattacharya et al. used a sperm-mediated shRNA delivery system to silence *MSTN* and its receptors (*ACVR2A* and *ACVR2B*), finding that silencing the *ACVR2B* receptor alone yielded the greatest increase in body weight and muscle fiber size [176].

Other production traits are also being targeted. To reduce abdominal fat, Park et al. used CRISPR/Cas9 in PGCs to knock out G0/G1 switch gene 2 (*G0S2*), a regulator of lipid metabolism and the resulting chickens showed significantly reduced abdominal adiposity and altered fatty acid profiles [177]. To address egg allergies, researchers have focused on the major allergens ovalbumin (*OVA*) and ovomucoid (*OVM*). Modifying the *OVM* protein to disrupt key structural bonds was shown to reduce its reactivity with IgE from allergic patients [178]. Building on this, Oishi et al. used CRISPR/Cas9 in PGCs to create chickens with knockout mutations in both the *OVA* and *OVM* genes, offering a path to producing hypoallergenic eggs [125].

Genome editing also provides a powerful solution to the economic and ethical challenges of sex selection in the layer industry. Eliminating male embryos before hatching would save significant costs. To this end, researchers have targeted genes involved in sex determination. Knocking out the androgen receptor (*AR*) gene resulted in testicular atrophy in males and undeveloped follicles in females, rendering both sexes infertile [179]. A more targeted approach focused on *DMRT1*, the key conserved gene for male development, which resides on the Z chromosome [27]. Using CRISPR/Cas9 in PGCs to create *DMRT1*-deficient chickens, Ioannidis et al. found that genetic males (Z^D+^/Z^D−^) underwent sex reversal and developed small ovaries, while genetic females (Z^D−^W) had complete gonadal dysgenesis, which underscores DMRT1’s critical dosage-dependent role in avian sex determination and opens new avenues for genetic control of sex [180]. By knocking out miR-2954 in chicken, Fallahshahroudi et al. demonstrated the resulting excess of transcripts in males was offset by the emergence of a highly targeted miR-2954-mediated transcript degradation mechanism during avian evolution [181]. These diverse applications in improving production traits are summarized in Table 4.

### 4.3. Development of Avian Models

Genome editing enables the creation of precisely engineered animal models to study gene function and to improve the efficiency of producing transgenic animals. For example, to create a platform for producing humanized antibodies, Schusser et al. used site-specific recombination to knock out the chicken immunoglobulin heavy chain (IgH) and light chain (IgL) genes in PGCs [182,183]. The resulting chickens were immunodeficient, lacking mature B cells, providing a valuable model for studying immunopathology and for future human antibody gene knock-in experiments [182,183]. To streamline future editing experiments, Rieblinger et al. created a transgenic chicken line that stably expresses Cas9, eliminating the need to deliver the Cas9 protein for each new experiment [184]. Targeting green fluorescent protein into the *NTN1* locus using CRISPR/Cas9 methodology, GFP localization faithfully replicated endogenous *NTN1* expression in the optic fissure and neural tube floorplate, which can be applied to a pertinent developmental context—coloboma [185].

These models are also invaluable for basic research. To investigate the function of the PRDM14 gene in birds, Hagihara et al. used CRISPR/Cas9 to knock it out in chicken PGCs. The resulting homozygous embryos died during early development, demonstrating that PRDM14 is essential for avian embryogenesis [186]. Recently, PGCs in *PRDM14* homozygous knockout chickens show reduced quantity and cannot be propagated in vitro, pointing to the multifunctional and essential role of PRDM14 during embryonic development [187]. In other avian models like the zebra finch, lentiviral vectors have been used to express human mutant huntingtin (mHTT) or to disrupt the CREB gene, creating powerful models for studying the neural mechanisms of Huntington’s disease and vocal learning, respectively [188,189].

A major challenge in producing genome-edited birds is the low efficiency of germline transmission, which is caused by competition between donor PGCs and the recipient embryo’s endogenous germ cells. To solve this, researchers have developed “surrogate host” or “sterile host” models. One strategy involves interspecific hybridization, such as crossing domestic fowl with guinea fowl, which produces sterile male hybrids that can act as PGC recipients, although this approach suffers from very low survival rates [190]. A more effective approach is to genetically ablate the host’s germ cells. Taylor et al. used TALENs to knock out the essential germ cell gene *DDX4* in PGCs. The resulting female chickens were completely sterile, providing an ideal recipient for transplantation of donor PGCs [120].

More advanced systems use inducible “suicide genes” to eliminate host germ cells on demand. Ballantyne et al. used CRISPR/Cas9 to knock in an inducible caspase-9 (iCaspase9) system into the *DAZL* locus, a key germ cell gene [191,192]. The iCaspase9 protein can be activated by a small-molecule drug, leading to targeted apoptosis of the host germ cells. Using these sterile hosts, the team successfully regenerated rare chicken breeds from cryopreserved PGCs and created new variants with edited feather traits [191,192]. A similar system developed by Chen et al. used the nitroreductase (*NTR*) enzyme, which converts the prodrug metronidazole into a cytotoxic agent, to ablate host germ cells by knocking the *NTR* gene into the *DDX4* locus [148]. The various avian models discussed in this section are detailed in Table 5.

### 4.4. Avian Bioreactors for Pharmaceutical Production

Chickens offer significant advantages as bioreactors for producing therapeutic proteins due to their rapid generation times, high egg output, and ability to perform complex post-translational modifications [193,194]. Early work used retroviral vectors to produce proteins like an anti-prion antibody (scFv-Fc), human granulocyte colony-stimulating factor (hG-CSF), and human urokinase (huPA) in the blood and/or eggs of transgenic chickens [104,195,196]. This approach was also used to create an oral immunotherapy agent for cedar pollen allergies by expressing engineered epitope peptides in egg whites, which showed therapeutic effects when fed to allergic mice [197]. A major advancement was the use of tissue-specific promoters, particularly the ovalbumin promoter, to restrict transgene expression to the oviduct. This allows for high-level protein production in egg whites while avoiding potential harm to the chicken. Using this strategy, researchers have produced human epidermal growth factor (EGF) [198], human erythropoietin (hEPO) [199], and the human antimicrobial peptide HNP4 in chicken eggs [200].

More recently, CRISPR-based targeted knock-in has replaced random integration methods, offering more precise and stable expression. Oishi et al. used CRISPR/Cas9 to knock the gene for human interferon-β (*hIFNβ*) directly into the ovalbumin locus in PGCs [201]. The resulting female chickens produced extremely high levels of hIFNβ in their egg whites (~3.5 mg/mL), creating an efficient production platform [201]. Similarly, Kim et al. and Yoo et al. used CRISPR to knock the gene for human adiponectin (*hADPN*), a therapeutic candidate for insulin resistance, into the ovalbumin locus [202,203]. The resulting hADPN produced in egg whites was shown to be biologically active and comparable to commercial standards, establishing the avian bioreactor as a viable platform for industrial-scale protein production [202,203]. A summary of these key studies, detailing the methods and therapeutic proteins produced, is provided in Table 6. The latest advancements in using chickens as oviduct bioreactors for producing protein-based drugs have been summarized in a recent comprehensive review [193].

## 5. Conclusions

**Figure 2:**
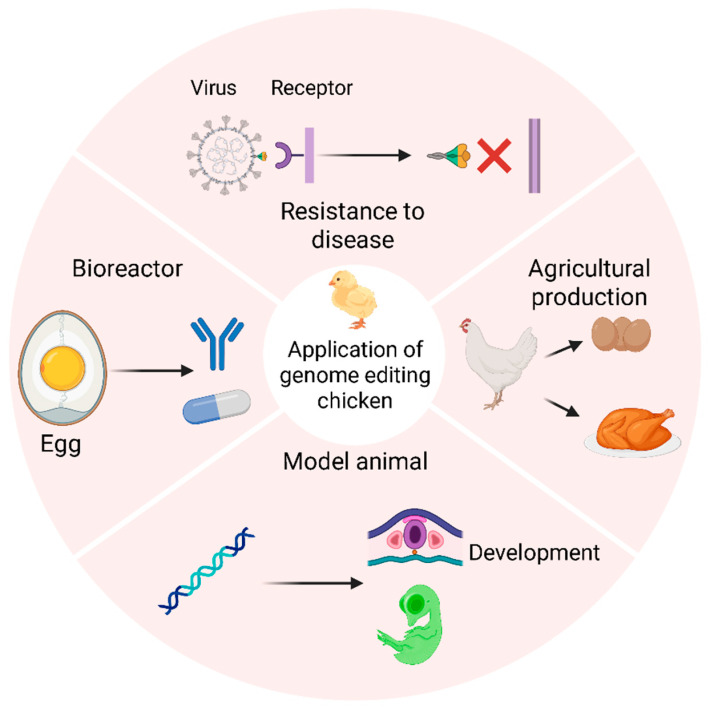
Major applications of genome editing in poultry. Genome editing technologies have enabled advancements across four major domains, many of which have been validated in both cell culture and live birds. These applications include engineering resistance to major pathogens, enhancing agricultural production traits, developing novel avian models for development research, and creating avian bioreactors for pharmaceutical production.

The potential applications of avian genome editing technologies are transformative for both agriculture and biomedical science. In agriculture, engineering resistance to devastating pathogens like avian influenza virus and Marek’s disease virus could revolutionize poultry health management, moving beyond a reliance on costly vaccination programs. Simultaneously, targeting genes like *MSTN* for muscle development or *DMRT1* for sex determination offers clear pathways to improving production efficiency. In parallel, avian models serve as invaluable platforms for studying gene function and human disease. The development of sterile surrogate hosts promises to streamline the production of these models, while the chicken’s oviduct can be harnessed as a highly efficient bioreactor for producing complex therapeutic proteins—a key advantage over mammalian systems (Figure 2).

Looking ahead, the future of avian genome editing will be defined by progress on two key frontiers. A critical priority is expanding PGC-based technologies beyond the chicken. The current reliance on this single model, while productive, represents a significant bottleneck. Successfully adapting these techniques to other commercially important birds like the turkey and duck, or to endangered species for genetic conservation, will unlock the full potential of this technology across a much broader range of agricultural and ecological applications.

In parallel, the industrial application of next-generation precision editing technologies, such as base and prime editors, offers transformative potential. These tools enable the creation of subtle, transgene-free genetic modifications, which may enhance public acceptance. More importantly, these tools can precisely install single-nucleotide variants without inducing double-strand breaks. This ability opens the door to sophisticated industrial strategies, including fine-tuning metabolic pathways for improved feed efficiency, enhancing climate resilience, and reducing protein allergenicity. As these next-generation systems mature and are deployed in a wider range of avian species, they will not only accelerate scientific discovery but also pave the way for a new era of sustainable, efficient, and resilient poultry production.

## Figures and Tables

**Table 1:** Core Signaling Pathways Regulating Avian PGC Self-Renewal and Survival.

Pathway	Key Ligands	Key Effectors	Principal Functions in PGCs
FGF/MAPK	FGF2	p-ERK1/2	Mitogenesis, Proliferation
PI3K/AKT/mTOR	Insulin, IGF-1, FGF2	p-AKT, p-mTOR	Survival, Anti-apoptosis, Metabolism, Proliferation
Activin/SMAD	Activin A	p-SMAD2/3	Maintenance of Self-Renewal
BMP/SMAD	BMP4	p-SMAD1/5/8	Germline Specification, Proliferation (non-clonal)
WNT	Wnt proteins	β-catenin	Modulation of Self-Renewal, Germline Specification

**Table 2:** Comparative analyses across different transfection methods.

Method	Protocol Specifics	Efficiency	References
Electroporation	With DMSO	80%	[84]
Electroporation	Without DMSO	26.5%	[84]
Electroporation	Percoll density gradients purified	16.6%	[85]
Electroporation	Unpurified	2.3%	[85]
Electroporation	Percoll density gradient purified	75.8%	[44]
Lipofection	Ammonium chloride-potassium	35.2%	[44]
Electroporation	Lonza system	71.13%	[86]
Lipofection	Lipofectamine™ 3000	1.38%	[86]
Lipofection	Transposon vector	67%	[87]
Lipofection	Heparin-free PGC medium	64.48%	[88]
Lipofection	Opti-MEM	19.56%	[88]
Lipofection	KO-DMEM	23.98%	[88]
Lipofection	GFP plasmid	<5%	[89]
Electroporation	GFP plasmid	80%	[89]
Adenovirus	ADV-GFP plasmid	83%	[89]

**Table 3:** Applications of poultry genome editing in resistance to disease.

Mediator	Method	Target Gene	Purpose	References
Embryo cells	lentiviral vector	*influenza A virus polymerase*	Resist to IVA	[161]
Embryo cells	lentiviral vector	*3D8 scFv*	Resist to IVA	[162,163]
PGCs	CRISPR/Cas9	*ANP32A*	Resist to IVA	[164]
PGCs	CRISPR/Cas9	*chNHE1 W38*	Resist to ALV-J	[166]
PGCs	CRISPR/Cas9	*chNHE1 W38*	Resist to ALV-J	[167]
PGCs	CRISPR/Cas9, Tol2 Transposon	*ICP4*	Resist to MDV	[170]

**Table 4:** Applications of poultry genome editing in agricultural production.

Mediator	Method	Target Gene	Purpose	References
Skeletal muscle	Adenovirus vector, CRISPR/Cas9	*MSTN*	Muscle production	[174]
Embryo cells	Adenovirus vector, CRISPR/Cas9	*MSTN*	Muscle production	[175]
Sperm	Lentiviral vector	*MSTN*, *ACVR2A*, *ACVR2B*	Muscle production	[176]
PGCs	CRISPR/Cas9	*G0S2*	Reduction in fat deposition	[177]
PGCs	CRISPR/Cas9	*OVA*, *OVM*	Egg production	[125]
PGCs	CRISPR/Cas9	*AR*	Sex determination	[179]
PGCs	CRISPR/Cas9	*DMRT1*	Sex determination	[180]
PGCs	CRISPR/Cas9	*miR-2954*	Sex differentiation	[181]

**Table 5:** Applications of poultry genome editing in model animals.

Mediator	Method	Target Gene	Purpose	References
PGCs	phiC31 integrase	*Ig H*	Model for disease analysis	[182]
PGCs	phiC31 integrase	*Ig L*	Model for disease analysis	[183]
PGCs	phiC31 integrase	*SpCas9*	Model for genome editing	[184]
PGCs	CRISPR/Cas9	*NTN1*	Model for disease analysis	[185]
PGCs	CRISPR/Cas9	*PRDM14*	Model for gene functional analysis	[186]
PGCs	TALEN, PiggyBac transposons	*PRDM14*	Model for gene functional analysis	[187]
Embryo cells	Lentiviral vectors	*CREB*	Model for disease analysis	[188]
Embryo cells	Lentiviral vectors	*HTT*	Model for disease analysis	[189]
PGCs	TALEN	*DDX4*	Model for sterility	[120]
PGCs	CRISPR/Cas9	*DAZL*, *DOW*, *FRZ*	Model for sterility	[192]
PGCs	CRISPR/Cas9	*DDX4*	Model for sterility	[148]

**Table 6:** Applications of poultry genome editing in bioreactors.

Mediator	Method	Target Proteins	Purpose	References
Embryo cells	Retrovirus vector	scFv-Fc	Bioreactor	[104]
Embryo cells	Retrovirus vector	hG-CSF	Bioreactor	[195]
Embryo cells	Retrovirus vector	huPA	Bioreactor	[196]
Embryo cells	Retrovirus vector	7Crp	Bioreactor	[197]
PGCs	PiggyBac transposons	EGF	Bioreactor	[198]
Embryo cells	Lentiviral vectors	hEPO	Bioreactor	[199]
Embryo cells	Lentiviral vectors	HNP4	Bioreactor	[200]
PGCs	CRISPR/Cas9	hIFN-β	Bioreactor	[201]
PGCs	CRISPR/Cas9	ADPN	Bioreactor	[203]

## Data Availability

No new data were created or analyzed in this study. Data sharing is not applicable to this article.
